# Measured and self-reported hypertension among women of reproductive age, Gambia, Kenya, Mozambique

**DOI:** 10.2471/BLT.24.292204

**Published:** 2025-05-26

**Authors:** Laura A Magee, Esperanca Sevene, Anne Rerimoi, Rachel Craik, Ashley Muteti, Marleen Temmerman, Marianne Vidler, Umberto D’Alessandro, Anna Roca, Jeffrey N Bone, Ash Sandhu, Marie-Laure Volvert, Hawanatu Jah, Salesio Macuacua, Angela Koech, Hiten D Mistry, Peter von Dadelszen, The PRECISE Network

**Affiliations:** a School of Life Course and Population Sciences, Faculty of Life Sciences and Medicine, King’s College London, Addison House, Guy’s Campus, Great Maze Pond, London, SE1 1UL, England.; b Centro de Investigação em Saúde de Manhiça, Manhiça, Mozambique.; c Zuri Nzilani Foundation, Nairobi, Kenya.; d Centre of Excellence in Women & Child Health, Aga Khan University, Nairobi, Kenya.; e Department of Obstetrics and Gynaecology and BC Children’s Hospital Research Institute, University of British Columbia, Vancouver, Canada.; f MRC Unit The Gambia at the London School of Hygiene & Tropical Medicine, Fajara, the Gambia.

## Abstract

**Problem:**

In sub-Saharan Africa, hypertension prevalence is usually estimated from participant recall. We assessed the accuracy of self-reported hypertension in women of reproductive age.

**Approach:**

In PRECISE (PREgnancy Care Integrating translational Science, Everywhere), an observational prospective cohort study, we recruited 1825 non-pregnant women of reproductive age, 610 in the Gambia, 609 in Kenya and 606 in Mozambique. We compared self-reported and measured hypertension (systolic blood pressure ≥ 140mmHg or diastolic blood pressure ≥ 90mmHg). We adjusted hypertension prevalence for age, body mass index, education, parity, and antihypertensive medicine and oral contraceptive use.

**Local setting:**

PRECISE was conducted in both urban and rural hospitals or clinics.

**Relevant changes:**

The women were generally in their late twenties and parous. Adjusted measured hypertension prevalence was higher in Mozambique (10.4%; 95% confidence interval, CI: 7.9–12.7) and the Gambia (9.3%; 95% CI: 6.6–12.6) than in Kenya (4.6%; 95% CI: 3.0–6.6). Self-reported hypertension prevalence was highest in the Gambia (12.9%; 95% CI: 10.2–15.9) versus Mozambique (4.2%; 95% CI: 2.8–5.7) or Kenya (6.7%; 95% CI: 5.0–8.6). Sensitivity of self-reported (versus measured) hypertension was less than 45% in all countries, with specificities more than 89%. Positive likelihood ratios were fair in the Gambia (3.70; 95% CI: 2.47–5.54), and good in Kenya (5.79; 95% CI: 3.36–9.98) and Mozambique (5.18; 95% CI: 2.56–10.46). All negative likelihood ratios were poor (≥ 0.20).

**Lessons learnt:**

Self-reported hypertension is unsuitable for population hypertension estimates among women of reproductive age in these countries.

## Résumé

**Problème:**

En Afrique subsaharienne, la prévalence de l’hypertension est généralement estimée à partir des souvenirs des participants. Nous avons évalué la précision de l’autodéclaration d’hypertension chez les femmes en âge de procréer.

**Approche:**

Dans le cadre de l’étude de cohorte prospective observationnelle PRECISE (PREgnancy Care Integrating translational Science, Everywhere (Soins pendant la grossesse intégrant des données de translation sur une zone étendue)), nous avons recruté 1825 femmes non enceintes et en âge de procréer: 610 en Gambie, 609 au Kenya et 606 au Mozambique. Nous avons comparé l’hypertension autodéclarée et l’hypertension mesurée (pression artérielle systolique ≥ 140 mmHg ou pression artérielle diastolique ≥ 90 mmHg). Nous avons ajusté la prévalence de l’hypertension en fonction de l’âge, de l’indice de masse corporelle, du niveau d’éducation, de la parité, de la prise d’antihypertenseurs et de contraceptifs oraux.

**Environnement local:**

L’étude PRECISE a été menée dans des hôpitaux ou des cliniques situés en zone urbaine et rurale.

**Changements significatifs:**

Les femmes étaient généralement âgées d’une vingtaine d’années et avaient déjà accouché au moins une fois. La prévalence de l’hypertension mesurée ajustée était plus élevée au Mozambique (10,4%; intervalle de confiance (IC) à 95%: 7,9–12,7) et en Gambie (9,3%; IC à 95%: 6,6–12,6) qu’au Kenya (4,6%; IC à 95%: 3,0–6,6). La prévalence de l’hypertension autodéclarée était la plus élevée en Gambie (12,9%; IC à 95%: 10,2–15,9) par rapport au Mozambique (4,2%; IC à 95%: 2,8–5,7) ou au Kenya (6,7%; IC à 95%: 5,0–8,6). La sensibilité de l’hypertension autodéclarée (par rapport à l’hypertension mesurée) était inférieure à 45% dans tous les pays, avec des spécificités supérieures à 89%. Les rapports de vraisemblance positifs étaient corrects en Gambie (3,70; IC à 95%: 2,47–5,54) et bons au Kenya (5,79; IC à 95%: 3,36–9,98) et au Mozambique (5,18; IC à 95%: 2,56–10,46). Tous les rapports de vraisemblance négatifs étaient faibles (≥ 0,20).

**Leçons tirées:**

L’autodéclaration ne convient pas pour estimer l’hypertension dans la population des femmes en âge de procréer dans ces pays.

## Resumen

**Situación:**

En África subsahariana, la prevalencia de hipertensión suele estimarse a partir del recuerdo de las participantes. Se evaluó la precisión de la hipertensión autoinformada en mujeres en edad reproductiva.

**Enfoque:**

En el estudio de cohorte prospectivo observacional PRECISE (PREgnancy Care Integrating translational Science, Everywhere; Atención al embarazo integrando ciencia traslacional en todas partes), se incluyeron 1825 mujeres no embarazadas en edad reproductiva: 610 en Gambia, 609 en Kenya y 606 en Mozambique. Se comparó la hipertensión autoinformada con la medida (presión arterial sistólica ≥140 mmHg o presión diastólica ≥90 mmHg). La prevalencia de hipertensión se ajustó por edad, índice de masa corporal, nivel educativo, paridad y uso de medicamentos antihipertensivos y anticonceptivos orales.

**Marco regional:**

El estudio PRECISE se realizó en hospitales o clínicas ubicadas en zonas urbanas y rurales.

**Cambios importantes:**

En general, las mujeres eran de finales de la veintena y ya habían tenido hijos. La prevalencia ajustada de hipertensión medida fue mayor en Mozambique (10,4%; intervalo de confianza, IC del 95%: 7,9-12,7) y en Gambia (9,3%; IC del 95%: 6,6-12,6) que en Kenya (4,6%; IC del 95%: 3,0-6,6). La prevalencia de hipertensión autoinformada fue más alta en Gambia (12,9%; IC del 95%: 10,2-15,9), en comparación con Mozambique (4,2%; IC del 95%: 2,8-5,7) y Kenya (6,7%; IC del 95%: 5,0-8,6). La sensibilidad de la hipertensión autoinformada frente a la medida fue inferior al 45% en los tres países, con especificidades superiores al 89%. Las razones de verosimilitud positiva fueron aceptables en Gambia (3,70; IC del 95%: 2,47-5,54) y buenas en Kenya (5,79; IC del 95%: 3,36-9,98) y Mozambique (5,18; IC del 95%: 2,56-10,46). Todas las razones de verosimilitud negativa fueron inadecuadas (≥0,20).

**Lecciones aprendidas:**

La hipertensión autoinformada no es adecuada para estimar la prevalencia de hipertensión a nivel poblacional en mujeres en edad reproductiva en estos países.

## ملخص

المشكلة عادةً ما يتم تقدير معدل انتشار ارتفاع ضغط الدم في جنوب الصحراء الكبرى الأفريقية، من خلال تذكر المشاركات. قمنا بتقييم دقة ارتفاع ضغط الدم المُبلّغ عنه ذاتيًا لدى النساء في سن الإنجاب.

الأسلوب إن PRECISE (رعاية الحمل: دمج العلوم الانتقالية في كل مكان) هي دراسة إترابية رصدية مستقبلية، قمنا فيها بإشراك 1825 امرأة غير حامل في سن الإنجاب، 610 منهن في غامبيا، و609 في كينيا، و606 في موزامبيق. قمنا بمقارنة ارتفاع ضغط الدم المُقاس والمُبلّغ عنه ذاتيًا (ضغط الدم الانقباضي ≥ 140 ملم زئبق، أو ضغط الدم الانبساطي ≥ 90 ملم زئبق). وقمنا بتعديل معدل انتشار ارتفاع ضغط الدم وفقًا للعمر، ومؤشر كتلة الجسم، والتعليم، وعدد مرات الولادة، واستخدام الأدوية الخافضة لضغط الدم، وموانع الحمل الفموية.

المواقع المحلية أُجريت دراسة PRECISE في كل من المستشفيات والعيادات الحضرية والريفية.

التغيّرات ذات الصلة كانت النساء عمومًا في أواخر العشرينات من أعمارهن وفي فترة حمل. كان معدل انتشار ارتفاع ضغط الدم المُعدّل والمقاس أعلى في موزمبيق (%10.4؛ بفاصل ثقة %95: 7.9 إلى 12.7) وغامبيا (%9.3؛ بفاصل ثقة %95: 6.6 إلى 12.6) عنه في كينيا (%4.6؛ بفاصل ثقة %95: 3.0 إلى 6.6). كان معدل انتشار ارتفاع ضغط الدم المبلغ عنه ذاتيًا هو الأعلى في غامبيا (%12.9؛ بفاصل ثقة %95: 10.2 إلى 15.9) مقابل موزامبيق (%4.2؛ بفاصل ثقة %95: 2.8 إلى 5.7) أو كينيا (%6.7؛ بفاصل ثقة %95: 5.0 إلى 8.6). كانت حساسية ارتفاع ضغط الدم المُبلّغ عنه ذاتيًا (مقارنةً بالقياس) أقل من %45 في جميع الدول، مع خصوصيات تتجاوز %89. كانت نسب الاحتمالية الإيجابية مقبولة في غامبيا (3.70؛ بفاصل ثقة %95: 2.47 إلى 5.54) وجيد في كينيا (5.79؛ بفاصل ثقة %95: 3.36 إلى 9.98) وموزامبيق (5.18؛ بفاصل ثقة %95: 2.56 إلى 10.46). وكانت جميع نسب الاحتمالية السلبية ضعيفة (≥ 0.20).

**الدروس المستفادة:**

ارتفاع ضغط الدم المُبلّغ عنه ذاتيًا غير مناسب لتقديرات ارتفاع ضغط الدم السكاني بين النساء في سن الإنجاب في هذه الدول.

## 摘要

**问题:**

在撒哈拉以南非洲地区，高血压患病率通常根据参与者的记忆予以估算。我们评估了育龄妇女中自述性高血压相关数据的准确性。

**方法:**

在 PRECISE (PREgnancy Care Integrating Translational Science, Everywhere) 研究——一项观察性前瞻性队列研究——中，我们共招募了 1,825 名处于育龄阶段但当前并未怀孕的妇女，其中冈比亚有 610 名，肯尼亚有 609 名，而莫桑比克有 606 名。我们对比了自述性和测量性高血压（收缩血压 ≥ 140 毫米汞柱或者舒张血压 ≥ 90 毫米汞柱）相关数据。我们按年龄、体重指数、受教育程度、生育数目以及降压药和口服避孕药使用情况调整了高血压患病率。

**当地状况:**

我们在城市和农村地区的医院或诊所都开展了 PRECISE 研究。

**相关变化:**

本次研究的对象主要为年龄将近 30 岁的经产妇女。对于调整后的测量性高血压患病率，莫桑比克【10.4%；95% 置信区间 (CI)：7.9-12.7】和冈比亚（9.3%；95% CI：6.6-12.6）比肯尼亚（4.6%；95% CI：3.0-6.6）高。对于自述性高血压患病率，与莫桑比克（4.2%；95% CI：2.8-5.7）或肯尼亚（6.7%；95% CI：5.0-8.6）相比，冈比亚（12.9%；95% CI：10.2-15.9）最高。这三个国家的自述性（对比测量性）高血压相关数据灵敏度均低于 45%，而特异度则超过了 89%。冈比亚（3.70；95% CI：2.47-5.54）的阳性似然比一般，而肯尼亚（5.79；95% CI：3.36-9.98）和莫桑比克（5.18；95% CI：2.56-10.46）的该比值较好。这三个国家的阴性似然比都很差 (≥ 0.20)。

**经验教训:**

自述性高血压相关数据并不适用于估算这三个国家育龄妇女中的高血压患病率。

## Резюме

**Проблема:**

В африканских странах южнее Сахары при оценке распространенности гипертензии обычно приходится полагаться на память пациента. Авторы оценили точность самостоятельных сообщений о гипертензии среди женщин репродуктивного возраста.

**Подход:**

В обсервационное проспективное когортное исследование PRECISE (PREgnancy Care Integrating translational Science, Everywhere, то есть «Ведение беременности на основе трансляционной медицины, глобальный охват») авторы включили 1825 небеременных женщин репродуктивного возраста: 610 в Гамбии, 609 в Кении и 606 в Мозамбике. Сравнивались измеренные данные о гипертензии и данные, которые сообщали сами женщины (систолическое давление ≥ 140 мм рт. ст., диастолическое ≥ 90 мм рт. ст.). Распространенность гипертензии была скорректирована по возрасту, индексу массы тела, образованию, наличию и количеству родов в анамнезе, а также по фактам приема антигипертензивных средств и оральных контрацептивов.

**Местные условия:**

Исследование PRECISE проводилось как в городских, так и в сельских больницах и клиниках.

**Осуществленные перемены:**

Женщины, как правило, были старше двадцати пяти лет, рожавшие. Согласно скорректированным данным измерений, распространенность гипертензии была выше в Мозамбике (10,4%, 95%-й ДИ: 7,9–12,7) и в Гамбии (9,3%, 95%-й ДИ: 6,6–12,6), нежели в Кении (4,6%, 95%-й ДИ: 3,0–6,6). Согласно самостоятельно сообщенным данным, гипертензия была наиболее распространена в Гамбии (12,9%, 95%-й ДИ: 10,2–15,9), нежели в Мозамбике (4,2%, 95%-й ДИ: 2,8–5,7) или в Кении (6,7%, 95%-й ДИ: 5,0–8,6). Чувствительность самостоятельного сообщения (по сравнению с измерениями) для гипертензии была ниже 45% во всех странах, а специфичность превышала 89%. Отношения правдоподобия для положительного результата исследования были среднего качества в Гамбии (3,70, 95%-й ДИ: 2,47–5,54) и хорошими в Кении (5,79, 95%-й ДИ: 3,36–9,98) и в Мозамбике (5,18, 95%-й ДИ: 2,56–10,46). Все отношения правдоподобия для отрицательного результата исследования были плохими (≥ 0,20).

**Выводы:**

Самостоятельные сообщения о гипертензии непригодны для оценки популяционной распространенности гипертензии среди женщин репродуктивного возраста в этих странах.

## Introduction

Hypertension is a leading and increasingly prevalent risk factor for cardiovascular disease. Accurate estimates of the prevalence of hypertension are needed for monitoring and evaluation of existing policies and programmes, to address cardiovascular disease. In sub-Saharan Africa, this information is sometimes collected through self-report in surveys. While the reliability of self-reported hypertension varies (e.g. by age, region and sex),^1^ the reliability in sub-Saharan Africa is unknown.

We sought to: (i) report the prevalence of hypertension in non-pregnant women of reproductive age, for whom cardiovascular disease prevention has particular potential; (ii) investigate the reliability of self-reported (versus measured) hypertension in such women; and (iii) compare self-reported hypertension prevalence between non-pregnant women of reproductive age and pregnant women in the Gambia, Kenya and Mozambique, within the PRECISE (PREgnancy Care Integrating translational Science, Everywhere) observational prospective cohort.^2^

## Local setting

Coordinated by King’s College London, United Kingdom of Great Britain and Northern Ireland, PRECISE was conducted in Kenya, Mozambique and the Gambia. PRECISE is an 8.8 million pounds sterling (£) project, with £2.0 million allocated for recruitment of women of reproductive age and completion of the first PRECISE visit.

PRECISE Gambia is led by the Medical Research Council Unit The Gambia at the London School of Hygiene & Tropical Medicine at one urban (Farafenni District Hospital; comprehensive maternity services) and two rural sites (Illiasa and Ngeyen Sanjal clinics; essential maternity services) in Farafenni District.

PRECISE Kenya is led by the Aga Khan University in two sites in Kilifi County: one urban (Mariakani Subcounty Hospital; comprehensive maternity services) and one rural (Rabai Subcounty Hospital; essential maternity services).

PRECISE Mozambique is led by the Centro de Investigação de Saúde de Manhiça, in two sites in Maputo Province: one urban (Manhiça District Hospital; comprehensive maternity services) and one rural (Xinavane Rural Hospital; comprehensive maternity services).

## Approach

We identified non-pregnant women of reproductive age (15–49 years) from family planning clinics (Mozambique, Kenya), or by random sampling from the local health and demographic surveillance system (the Gambia). Pregnant women of reproductive age were recruited during antenatal care. All provided written, informed consent. The study was approved by the Research Ethics Boards at King’s College London, United Kingdom (HR-17/18–7855) and the University of British Columbia, Canada (H18–02828), and within each country (2018/REC-74, Kenya; 545/CNBS/18, Mozambique; SCC 1619, the Gambia).

To determine self-reported hypertension, women were asked, “Have you ever been told by a doctor or other health worker that you have high blood pressure?” Then, blood pressure was measured at least twice using a semi-automated oscillometric device,^3^ after 5 minutes’ rest, having removed restrictive arm clothing and checking cuff size and patient position (feet on floor, arm at heart level). For each systolic and diastolic blood pressure measurement, when the first and second readings were within 10 mmHg, we used the average. If the first and second readings were > 10 mmHg different, a third reading was taken and we used the average of the second and third readings.^4^ We defined hypertension as systolic blood pressure ≥ 140 mmHg or diastolic blood pressure ≥ 90 mmHg.^5^ We referred women with hypertension for relevant care.

For measured and self-reported hypertension, we used multivariable logistic regression to estimate country-specific hypertension prevalence, after adjustment for age, body mass index (BMI), basic education, parity (nulliparous or parous), antihypertensive medicine use (any or none) and oral contraceptives use in the preceding 12 months (before pregnancy in pregnant women of reproductive age). We adjusted country prevalence of hypertension using model-predicted probabilities averaged over adjustment factors. These values are equivalent to the expected prevalence per country, assuming each had the same baseline characteristics for which adjustment was made. We calculated confidence intervals (CIs) by non-parametric bootstrapping, estimated by the delta method, given sparse data and convergence in some bootstrap samples.

Among the non-pregnant women, we assessed accuracy of self-reported versus measured hypertension by calculating: (i) sensitivity (proportion who self-reported hypertension, among women with measured hypertension); (ii) specificity (proportion who self-reported no hypertension, among women with normal measured blood pressure); and (iii) positive and negative likelihood ratios. The positive likelihood ratio indicates how much the odds of measured hypertension increase when hypertension is self-reported, calculated as (sensitivity)/(1–specificity). The negative likelihood ratio indicates how much the odds of measured hypertension decrease when hypertension is self-reported, calculated as (1–sensitivity)/(specificity). A good negative likelihood ratio is < 0.20 and a good positive likelihood ratio is ≥ 5.0.^6^^–^^8^

Within each country, we compared the prevalence of self-reported hypertension between the non-pregnant and pregnant women by calculating risk difference and 95% bootstrap CIs.

We used R, version 4.2.1 (R Foundation, Vienna, Austria) for analyses.

## Relevant changes

From June 2019 to December 2022, we recruited 1825 non-pregnant women of reproductive age and 6770 pregnant women (respectively, 609 and 3450 in Kenya; 606 and 2097 in Mozambique; and 610 and 1223 in the Gambia). There were between-country differences in baseline characteristics for both non-pregnant and pregnant women (Table 1). The non-pregnant women of reproductive age were in their late twenties, while pregnant women were in their early-to-mid-twenties. Non-pregnant and pregnant Gambian women had lower BMI, with a quarter of each group of women being underweight. Most Kenyan and Mozambican women had at least primary education, whereas most Gambian women had no basic education. Few women reported a history of kidney disease or diabetes. Oral contraceptive use in the preceding 12 months was low in the Gambia (3 women; 0.5%) and Kenya (12 women; 2.0%), but more than 10% in Mozambique (98 women; 16.2%).

**Table 1:** Baseline characteristics of the women of reproductive age, by pregnancy status, the Gambia, Kenya and Mozambique, June 2019 to December 2022

Characteristic	Not pregnant				Pregnant		
	Gambia (*n* = 610)	Kenya (*n* = 609)	Mozambique (*n* = 606)		Gambia (*n* = 1223)	Kenya (*n* = 3450)	Mozambique (*n* = 2097)
**Age in years, median (IQR)**	28.0 (22.0–36.0)	27.0 (23.0–33.0)	28.0 (23.0–34.8)		26.0 (22.0–31.0)	26.0 (23.0–31.0)	23.0 (19.0–29.0)
Missing	11 (NA)	0 (NA)	0 (NA)		4 (NA)	1 (NA)	0 (NA)
**Body mass index in kg/m**²**, no. (%)**							
< 18.5	153/609 (25.1)	53/601 (8.8)	22/597 (3.7)		163/1209 (13.5)	143/3386 (4.2)	39/2070 (1.9)
18.5–24.9	328/609 (53.9)	373/601 (62.0)	357/597 (59.8)		770/1209 (63.7)	1827/3386 (54.0)	1154/2070 (55.7)
25.0–29.9	85/609 (14.0)	120/601 (20.0)	150/597 (25.1)		202/1209 (16.7)	894/3386 (26.4)	652/2070 (31.5)
≥ 30.0	43/609 (7.0)	55/601 (9.2)	68/597 (11.4)		74/1209 (6.1)	522/3386 (15.4)	225/2070 (10.9)
Missing	1 (NA)	8 (NA)	9 (NA)		14 (NA)	64 (NA)	27 (NA)
**Religion, no. (%)**							
Christian	8/609 (1.3)	379/606 (62.5)	550/605 (90.9)		8/1222 (0.7)	2082/3436 (60.6)	2042/2095 (97.5)
Muslim	601/609 (98.7)	222/606 (36.6)	11/605 (1.8)		1214/1222 (99.3)	1343/3436 (39.1)	27/2095 (1.3)
Traditional, spiritualist, animist	0/609 (0.0)	1/606 (0.2)	35/605 (5.8)		0/1222 (0.0)	4/3436 (0.1)	11/2095 (0.5)
Buddhist	0/609 (0.0)	0/606 (0.0)	0/605 (0.0)		0/1222 (0.0)	0/3436 (0.0)	2/2095 (0.1)
Other	0/609 (0.0)	0/606 (0.0)	5/605 (0.8)		0/1222 (0.0)	1/3436 (0.0)	3/2095 (0.1)
None	0/609 (0.0)	4/606 (0.7)	4/605 (0.7)		0/1222 (0.0)	6/3436 (0.2)	10/2095 (0.5)
Missing	1 (NA)	3 (NA)	1 (NA)		1 (NA)	14 (NA)	2 (NA)
**Education** ^a^ **, no. (%)**							
None	373/610 (61.1)	62/606 (10.2)	65/606 (10.7)		781/1222 (63.9)	322/3437 (9.4)	109/2097 (5.2)
Primary	71/610 (11.7)	367/606 (60.6)	266/606 (43.9)		193/1222 (15.8)	1812/3437 (52.7)	704/2097 (33.6)
Secondary	119/610 (19.5)	116/606 (19.1)	271/606 (44.7)		192/1222 (15.7)	895/3437 (26.0)	1258/2097 (60.0)
Higher	47/610 (7.7)	61/606 (10.1)	4/606 (0.7)		56/1222 (4.6)	408/3437 (11.9)	26/2097 (1.2)
Missing	0 (NA)	3 (NA)	0 (NA)		1 (NA)	13 (NA)	0 (NA)
**Parity, no. (%)**							
Nulliparous	175/610 (28.7)	39/609 (6.4)	60/606 (9.9)		234/1223 (19.1)	990/3450 (28.7)	828/2097 (39.5)
Parous	435/610 (71.3)	570/609 (93.6)	546/606 (90.1)		989/1223 (80.9)	2460/3450 (71.3)	1269/2097 (60.5)
**Self-reported hypertension, no. (%)**							
No	522/608 (85.9)	551/603 (91.4)	569/606 (93.9)		1063/1220 (87.1)	3202/3429 (93.4)	2044/2095 (97.6)
Yes	83/608 (13.7)	49/603 (8.1)	29/606 (4.8)		153/1220 (12.5)	213/3429 (6.2)	44/2095 (2.1)
Don’t know	3/608 (0.4)	3/603 (0.5)	8/606 (1.3)		4/1220 (0.4)	14/3429 (0.4)	7/2095 (0.3)
Missing	2 (NA)	6 (NA)	0 (NA)		3 (NA)	21 (NA)	2 (NA)
**Taking antihypertensive medicine, no. (%)**	4/83 (4.8)	9/49 (18.4)	1/29 (3.4)		6/153 (3.9)	15/213 (7.0)	0/44 (0.0)
**Chronic kidney disease, no. (%)**							
No	605/608 (99.5)	592/603 (98.2)	591/606 (97.5)		1207/1220 (98.9)	3375/3428 (98.5)	2085/2096 (99.5)
Yes	2/608 (0.3)	6/603 (1.0)	1/606 (0.2)		7/1220 (0.6)	29/3428 (0.8)	1/2096 (0.0)
Don’t know	1/608 (0.2)	5/603 (0.8)	14/606 (2.3)		6/1220 (0.5)	24/3428 (0.7)	10/2096 (0.5)
Missing	2 (NA)	6 (NA)	0 (NA)		3 (NA)	22 (NA)	1 (NA)
**Diabetes, no. (%)**							
No	599/608 (98.5)	595/603 (98.6)	599/605 (99.0)		1208/1220 (99.0)	3380/3429 (98.5)	2087/2095 (99.7)
Yes	7/608 (1.2)	4/603 (0.7)	0/605 (0.0)		8/1220 (0.7)	30/3429 (0.9)	3/2095 (0.1)
Do not wish to answer	2/608 (0.3)	4/603 (0.7)	6/605 (1.0)		4/1220 (0.3)	19/3429 (0.6)	5/2095 (0.2)
Missing	2 (NA)	6 (NA)	1 (NA)		3 (NA)	21 (NA)	2 (NA)
**Oral contraceptive use in previous 12 months^b^, no. (%)**	3/609 (0.5)	12/609 (2.0)	98/606 (16.2)		11/1222 (0.9)	61/3449 (1.8)	256/2097 (12.2)
Missing	1 (NA)	0 (NA)	0 (NA)		1 (NA)	1 (NA)	0 (NA)

BMI: body mass index; IQR: interquartile range; NA: not applicable.

^a^ We defined basic education as any formal schooling at primary, secondary or post-secondary school-level.

^b^ Among unselected pregnant women, this use was the 12 months before pregnancy.

Among the non-pregnant women of reproductive age, adjusted prevalence rates for measured hypertension were higher in the Gambia (9.3%; 95% CI: 6.6 to 12.6) and Mozambique (10.4%; 95% CI: 7.9 to 12.7) than Kenya (4.6%; 95% CI: 3.0 to 6.6; Table 2). For self-reported hypertension, adjusted prevalence rates were higher in the Gambia (12.9%; 95% CI: 10.2 to 15.9) than Kenya (6.7%; 95% CI: 5.0 to 8.6) and Mozambique (4.2%: 95% CI: 2.8 to 5.7). Within each country, self-reported hypertension had low sensitivity (< 45%) and high specificity (≥ 89%) compared with measured hypertension. In all countries, the negative likelihood ratios were poor (above 0.65 in all countries), suggesting that self-reported hypertension cannot provide reassurance about the presence of measured hypertension. In Kenya and Mozambique, the positive likelihood ratio indicated an increased likelihood of measured hypertension with self-reported hypertension. The pattern was consistent across baseline characteristics of age, BMI and basic education; due to small sample sizes, we could not examine the effect on sensitivity and specificity of parity, use of antihypertensives or use of oral contraceptives.

**Table 2:** Measured and self-reported hypertension among women of reproductive age, the Gambia, Kenya and Mozambique, June 2019 to December 2022

Outcome	Gambia	Kenya	Mozambique
**Non-pregnant women of reproductive age**	*n* = 610	*n* = 609	*n* = 606
Measured hypertension^a^, % (95% CI)	9.3 (6.6 to 12.6)^b^	4.6 (3.0 to 6.6)^b^	10.4 (7.9 to 12.7)
Self-reported hypertension, % (95% CI)^a^	12.9 (10.2 to 15.9)^b^	6.7 (5.0 to 8.6)^b^	4.2 (2.8 to 5.7)
**Pregnant women**	*n* = 1223	*n* = 3450	*n* = 2097
Self-reported hypertension^a^, % (95% CI)	12.9 (10.9 to 15.4)^b^	6.0 (5.3 to 6.8)	2.7 (1.9 to 3.5)^b^
**Non-pregnant women of reproductive age: self-reported versus measured hypertension**			
Sensitivity, % (95% CI)	41 (28 to 56)	38 (21 to 56)	17 (9 to 29)
Specificity, % (95% CI)	89 (86 to 91)	94 (91 to 95)	97 (95 to 98)
Negative likelihood ratio (95% CI)	0.66 (0.53 to 0.83)	0.67 (0.51 to 0.87)	0.86 (0.77 to 0.96)
Positive likelihood ratio (95% CI)	3.70 (2.47 to 5.54)	5.79 (3.36 to 9.98)	5.18 (2.56 to 10.46)
**Self-reported hypertension: non-pregnant women of reproductive age versus pregnant women **			
Adjusted risk difference, % (95% CI)	0.0 (−3.5 to 3.3)	0.6 (−1.3 to 2.9)	1.4 (−1.6 to 2.9)

CI: confidence interval.

^a^ We adjusted hypertension prevalence for age, body mass index, basic education, parity, antihypertensive medication and history of taking oral contraceptives in the previous 12 months (and specifically before pregnancy for pregnant women).

^b^ Data were missing for three women in Kenya and two in the Gambia.

Among pregnant women, the adjusted prevalence of self-reported hypertension was lowest in Mozambique (2.7%; 95% CI: 1.9 to 3.5) and highest in the Gambia (12.9%; 95% CI: 10.9 to 15.4), as it was also for the non-pregnant women of reproductive age (Table 2). Adjusted rates of self-reported hypertension did not differ between the non-pregnant women of reproductive age and pregnant women.

## Lessons learnt

Our study showed that measured hypertension prevalence among non-pregnant women of reproductive age, after adjusting for participant characteristics, were higher in Mozambique and the Gambia than Kenya, and lower than published estimates, about 30.0% in Mozambique, 17.0% in the Gambia and 11.0% in Kenya.^9^^–^^11^ However, the populations recruited in PRECISE may not be representative of national populations. The women in our study were recruited from selected districts and in different ways. In the Gambia, for example, a quarter of the women were underweight (versus 7.0% at national level),^9^ and < 1% had known risk factors for hypertension (other noncommunicable diseases). In Kenya, national hypertension prevalence rates are not disaggregated by age, so rates could be higher than in our study. Mozambican women in our study are particularly active, with manual labour jobs (e.g. sugar cane farm work), hence our sample may be different from a national one.^12^^,^^13^ As most women were parous and few women were taking antihypertensives or oral contraceptives, we could not adjust for these factors. Therefore, further investigation is needed in nationally representative samples.

We assessed the accuracy of self-reported hypertension versus measured hypertension. Despite the simplicity and widespread use of self-reported hypertension in sub-Saharan Africa, our results show that this method is insensitive for detecting measured hypertension and unsuitable for population hypertension estimates among women of reproductive age (Box 1).

Box 1 **Summary of main lessons learnt**

- We found that self-reported hypertension is specific, but not sensitive, for measured hypertension, making self-report an inadequate screening test.
- Our findings support the World Health Organization’s STEPwise approach to noncommunicable disease risk-factor surveillance,^14^ which is to include self-reported and biomedical measurements of blood pressure.

Our findings align with a systematic review that found that self-reporting underestimated hypertension prevalence.^1^ While less than half of people with hypertension would have been diagnosed based on self-report (sensitivity: 42.1%; specificity: 89.5%), there was substantial variation in diagnostic accuracy of self-reported (versus measured) hypertension across countries and age groups.^1^ Only one study included a sub-Saharan Africa country (Ghana), in which self-report performed poorly (sensitivity: 13.0%; specificity: 97.0%) in a population with mean age of 60 years.^1^ Other studies have found reliability of self-reported hypertension to be context-specific.^6^^,^^7^

Our findings have implications for health and demographic surveillance systems, and provide evidence in young African women to support the approach recommended in the Global Monitoring Framework for noncommunicable diseases (STEPwise), to include blood pressure measurement by standardized methods, alongside self-reporting (Box 1).^14^ Of note, our blood pressure measurements reflected population screening recommendations, which differ from clinical practice guidelines for diagnosis of hypertension in individuals (that is, repeat blood pressure measurement on more than two separate occasions).^5^
